# Current Concepts and Controversies in Orthopaedic Oncology

**DOI:** 10.2174/1874325001711010478

**Published:** 2017-05-31

**Authors:** Vincent Y. Ng

**Affiliations:** Department of Orthopaedics Greenebaum Cancer Center, University of Maryland Medical Center, 110 S. Paca St., 6^th^ Floor, Suite 300, Baltimore, MD 21201, USA

The manuscripts in this thematic issue on musculoskeletal oncology present different techniques and address difficult topics that have received limited attention in the current literature. Because of the wide variety of clinical situations encountered with bone and soft tissue tumors, it is essential for oncologic surgeons to be both facile and versatile. The following manuscripts contain valuable techniques for readers to augment their surgical armamentarium and clinical acumen.

In the first article, Bawa *et al.* discuss the unique technique of thorascopic-assisted resection of a chondrosarcoma in a pediatric patient. Chondroid lesions can be a diagnostic challenge. A systematic review of malignant degeneration of synovial chondromatosis is discussed by Ng *et al.* In the third article, Conrad *et al.* discuss their experience with treating unicameral bone cysts.

The remainder of articles focuses on techniques and issues related to surgical resection and reconstruction. The accuracy and precision of surgical navigation is explored by Gundle *et al.* Infection and wound healing can be a significant challenge. Gupta *et al.* explores using negative pressure wound therapy and also a novel antibiotic spacer to address these issues.

We hope that orthopaedic oncologists and general practitioners alike find this special issue both useful and interesting.

